# Khat Induced Toxicity: Role on Its Modulating Effects on Inflammation and Oxidative Stability

**DOI:** 10.1155/2018/5896041

**Published:** 2018-05-30

**Authors:** Siddig Ibrahim Abdelwahab, Rashad Alsanosy, Manal Mohamed Elhassan Taha, Syam Mohan

**Affiliations:** ^1^Substance Abuse Research Centre, Jazan University, Jazan, Saudi Arabia; ^2^Medical Research Centre, Jazan University, Jazan, Saudi Arabia

## Abstract

Long-term khat (*Catha edulis* Forsk.) chewing has negative effects on human body. Khat constituents appear to be capable of disturbing the delicate equilibrium between damaging and protective mechanisms of a cell that is essential for optimal activity, thereby producing oxidative damage. Therefore, the current study was designed to understand the role of khat on cell toxicity, oxidative stability, and inflammation. Khat was extracted using 60% methanol and assessed calorimetrically for its phenolic and flavonoid contents. 1,1-diphenyl-2-picrylhydrazyl (DPPH) radical scavenging, oxygen radical absorbance capacity (ORAC), and ferric reducing/antioxidant power (FRAP) assays were used to assess the antioxidant properties. Lipopolysaccharide and interferon gamma induced murine monocytic macrophages cell line (RAW 264.7) were used to assess khat effects on cellular inflammation, oxidative stability, and viability. Khat possesses high content of polyphenols and flavonoids. The results showed a strong potency of antioxidants in DPPH, ORAC, and FRAP assays. Khat decreases the production of the proinflammatory nitric oxide and induces cytotoxicity and reactive oxygen species inhibition. Heavy khat consumption induced-toxicity and symptoms are probably due the harmful effects of its polyphenolic contents.

## 1. Introduction

*Catha edulis* is an evergreen plant belonging to the Celastraceae family. Locally it is known in different names such as khat, qat, and gat in Yemen, qaat and jaad in Somalia, and chat in Ethiopia [1]. It is mainly cultivated in Africa and Arabian Peninsula. Those who use this plant chew the fresh leaves for pleasurable and stimulating effect. It is chewed alone and sometimes together with cigar and alcohol [2]. The prevalence of khat chewing is widespread even in USA and Europe due to the migration of local people from Yemen, Somalia, Eritrea, and Saudi Arabia [3].

There are many health hazards associated with the consumption of khat. It literally affects every human organ systems and induces adverse effects as per literatures. In the central nervous system it produces euphoria and mild excitement, which are later gradually replaced by mild dysphoria, anxiety, insomnia, and anorexia [4]. The CNS effects of khat have been believed due to the chemical content cathinone, which is having closely similar structure of amphetamine, a known psychostimulant [5]. Hyperactivity and logorrhea also have been reported with khat consumption. In some case reports, the khat consumption has been found to induce schizophreniform psychosis and paranoid psychosis secondary to other psychiatric disorders [6]. In the cardiovascular system, khat has been shown to induce acute myocardial infarction and coronary heart failure and ischemic conditions [7]. One of the studies suggests the elevation of systolic blood pressure in khat consumers due to stimulant effect in *β*1 adrenoceptor in heart [7]. Khat has showed stomatitis, esophagitis, and gastritis in the GIT. Apart from this, anorexia and constipation are frequent with heavy users of khat [8].

Regarding the toxicity, it is not yet found any proper link between any mechanism and toxicity by khat. The available research focused on toxicity mainly due to the content of cathine and other phytochemicals [9]. But above all of this, one such property is very visible in previous research, and it is the generation of antioxidant in khat plant. Antioxidants are believed to be a safe property in generally. But it is to be noted that this safe compound has a potential to be harm in some situation or in certain amount [10]. For instance Vitamin E, *β*-Carotene, and Lipoic acid are well known antioxidants. But reports suggest that they have toxic potential as well. Vitamin E (Tocopherols) has been found to be involved in reduction of transition state metals, eventually leads to formation of free radicals [11]. It is well known for increased fatal myocardial infarction. Reference [12] found that, due to its oxidisability, it would act as a sensor of oxidative stress rather than a direct antioxidant. Similarly, another known antioxidant, *β*-carotene, resulted in an 18% increase in lung cancer incidence [13].

In the light of these facts, we believe and propose that the toxicity exerted by khat may be due to the prooxidant activity of high antioxidant constituents present in it. In our previous research, khat's adverse effects were proven* in vivo *and* in vitro *[14–16]. The role of exposure to excess antioxidant in hepatocytes* in vitro* and in vivo model was proven in our previous paper [14, 15]. Moreover, significant increase of reactive oxygen species and apoptosis (programmed cell death) was also noticed in kidney and cardiac cells with khat treatment [16, 17]. Extensive use of khat among local population and our previous findings led us to explore the khat modulating effect on inflammation and oxidative stability. Therefore, the current study was designed to understand the role of khat on cell toxicity, oxidative stability, and inflammation.

## 2. Materials and Methods

### 2.1. Extraction

*Catha edulis* leaves were obtained from Substance Abuse Research Centre, Jazan University, under a license from the Ministry of Interior, Saudi Arabia. The fresh bundles were transported to the laboratory and store immediately in −80°C. Extraction was performed according to the method described earlier [18]. Shade-dried and ground leaves of khat were extracted successively with 60% methanol (3 × 300 mL) for two days at room temperature to obtain crude methanolic (hydroalcoholic) extract; the extracts were pooled and the solvent was evaporated using rotary evaporator (Buchi, Flawil, Switzerland). The extract was kept at 4°C for further analyses.

### 2.2. Total Phenolic and Flavonoid Contents

The total phenolic and flavonoid contents of the extract were determined by the Folin–Ciocalteu and aluminum chloride colorimetric methods, respectively [19]. Extract was dissolved in methanol and the absorbance was obtained spectrophotometrically. Findings were expressed as the corresponding standards used in the study.

### 2.3. 1,1-Diphenyl-2-picrylhydrazyl (DPPH) Radical Scavenging Activity of Khat

The DPPH scavenging activity of khat extract has been done by using a method described earlier with slight modification [20]. In this study, the concentration of khat extract was kept at 0–50 0–50 *μ*g/mL in methanol. Briefly the test solution was made by adding 1 mL of 0.3 mM DPPH ethanol solution with 2.5 mL of sample solution. Meanwhile the blank was constituted by 2.5 mL sample with 1 mL of methanol alone. One milliliter of DPPH added with 2.5 mL methanol was used as negative control (blank). Positive control used in this assay was ascorbic acid. All these solution were made and kept at room temperature for a while to react for 30 minutes in dark, followed by the fact that absorbance values were recorded at 518 nm. The values were converted into percentage antioxidant activity using the following formula:

(1) $$\begin{array}{c} \%\text{ Inhibition}=\left[\;\frac{\left(A_{B}-A_{A}\right)}{A_{B}}\;\right]\times 100, \end{array}$$

where *A*_*B*_ is absorption of blank sample and *A*_*A*_ is absorption of tested samples.

The IC_50_ as well as the kinetics of DPPH scavenging activity was determined.

### 2.4. ORAC Antioxidant Activity Assay

The antioxidant activity assay was done using ORAC (oxygen radical absorbance capacity) assay as described earlier slight modifications [21]. Briefly 175 *μ*L of the samples/blank was dissolved with PBS at concentrations of 160 *μ*/gml, pH 7.4. 75 mMTrolox was used to prepare serial dilutions. To perform the assay, 25 *μ*l of khat sample, Trolox (the standard), and positive control/PBS (blank) were added to the wells of 96-well black microplates. To each well, 150 *μ*l of fluorescent sodium salt solution was added. The plates were then incubated for 45 minutes at 37°C. The wells were added with 25 *μ*l of 2,20-azobis (2-amidinopropane) dihydrochloride (AAPH) solution to a volume of 200 *μ*l/well. The fluorescence emission was recorded till the values become zero at 485 nm excitation and 535 emission wavelength using Perkin–Elmer LS 55 fluorescence spectrophotometer at 37°C. Data were collected every 2 mins for a duration of 2 hrs and were analyzed by calculating the differences of areas under the fluorescein decay curve (AUC) between the blank and the sample. AUC was calculated for the sample, standard, and the positive control from the equation

(2) AUC=1+RFU1RFU0+RFU2RFU0+RFU3RFU0+⋯+RFU119RFU0+RFU120RFU0,RFU0=relative  fluorescence  unit  value  of  time  point  zero,

RFU_x_ is relative fluorescence unit value of time points (e.g., RFU_2_ is relative fluorescence unit at minute two). Next, the Net AUC was calculated by subtracting the Blank AUC from the AUC of each sample, the standards, and the positive control. Final ORAC values were expressed as the equivalent concentration of Trolox (TE) that gives the same level of antioxidant activity as the samples at 100 *μ*g/mL.

### 2.5. Ferric Reducing/Antioxidant Power (FRAP) Assay

Ferric reducing/antioxidant power assay (FRAP) was conducted according to the method of Benzie and Strain with slight modification [22]. The stock solution for the assay was prepared by 300 mM acetate buffer (pH 3.6) and 10 mM TPTZ (2, 4, 6-tripyridyl-s-triazine) solution in 40 mMHCl and 20 mM FeCl3·_6_H_2_O solution. The working solution was prepared freshly using acetate buffer (25 ml), 2.5 ml TPTZ, and 2.5 ml FeCl3·6H2O. The experiment was conducted in a constant temperature of 37°C. 190 *μ*L of the FRAP solution was then allowed to react with 10 *μ*L extract for 30 minutes in dark. The production of ferrous tripyridyltriazine complex was observed and readings were noted using spectrophotometer at 593 nm. The standard curve was linear between 200 and 1000 *μ*M FeSO_4_. Results are expressed as *μ*MFe(II)/g dry mass and compared with those of quercetin.

## 3. Role of Khat on Inflammation

### 3.1. Chemical

The reagents and consumables were obtained from commercial suppliers. Briefly, DMEM media (Flowlab™, Australia), penicillin streptomycin antibiotics (Flowlab™, Australia), fetal bovine serum (FBS; iDNA technologies Inc., Singapore), recombinant mouse IFN-*γ* (eBioscience Inc., USA), lipopolysaccharide from* Escherichia coli* (strain 055:B5), sulphanilamide, naphtyethyenediamine anddiphenylpicrylhydrazine (DPPH; Sigma Aldrich, USA); and 3-(4,5-dimethylthiazol-2-yl)-2,5-diphenyl tetratzolium bromide (MTT; FlukaChemie GmbH, Switzerland).

### 3.2. Cell Culture and Stimulation

RAW 264.7 cells (murine monocytic macrophages) were purchased from ATCC. The cells were maintained in cell culture media (DMEM) added with 10% fatal bovine serum and 1% antibiotics (penicillin streptomycin) at 37°C and 5% CO_2_. The cell culture was maintained always at 80% confluency, and the viability of the cells was assured to above 90% using trypan blue exclusion assay. Cell were seeded into the 96-well plates in at 4 × 10^5^ cells/well at 50 *μ*L volume per well. The seeded cells were incubated for 2 hours at 37°C, 5% CO_2_ for the attachment of the cells. 100 U/ml of IFN-*γ* and 5 *μ*g/ml of LPS were then added to the well to stimulate with or without the extracts. The final volume was made in to 100 *μ*L per well. Control wells were added with 0.1% DMSO. Cells were further incubated at 37°C, 5% CO_2_ for 17–20 h. The culture supernatant was subjected to Griess assay for nitrite determination and the cells remaining in the well were tested for cell viability assay by using MTT reagent.

### 3.3. Nitrite Determination

To evaluate the inhibitory activity of plant extract or compound on nitric oxide (NO) production, culture media was assayed using Griess reaction. Briefly, an equal volume of Griess reagent (1% sulphanilamide and 0.1% N-(l-naphthyl)-ethylene diamine dihydrochloride, dissolved in 2.5% H_3_PO_4_) was mixed with culture supernatant and colour development was measured at 550 nm using a microplate reader (SpectraMax Plus, Molecular Devices Inc., Sunnyvale, CA, USA). The amount of nitrite in the culture supernatant was calculated from a standard curve (0–100 *μ*M) of sodium nitrite freshly prepared in deionized water. Percentage of the NO inhibition was calculated by using nitrate level of IFN-*γ*/LPS-induced group as the control.

(3) NO  inhibitory  %=NO2 −control−NO2 −sampleNO2 −control×100%.

### 3.4. Cell Viability

The cytotoxicity of the khat on cultured cells was determined by assaying the reduction of MTT reagents to formazan salts. After removing of supernatant, the MTT reagents (0.05 mg/ml dissolved in sterile PBS, pH 7.0) were added to each well. The cells remaining were incubated at 37°C for 4 h and the formazan salts formed were dissolved by adding 100 *μ*L of 100% DMSO in each well. The absorbance was then measured at 570 nm using SpectraMax Plus microplate reader (Molecular Devices, USA). The percentage of cell viability was calculated by using the cell viability of IFN-*γ*/LPS-induced group as the control.

(4) $$\begin{array}{c} \text{Cell Viability }\left(\;\%\;\right)=\frac{{OD}_{control}-{OD}_{sample}}{{OD}_{control}}\times 100\%. \end{array}$$

### 3.5. Reactive Oxygen Species (ROS) Assay

The ability of khat to induce intracellular ROS formation was determined using DCFH-DA (2′,7′-dicholorofluorescein diacetate) assay by fluorescence spectrophotometry. In brief, RAW cells were treated with 4 mg/mL of khat for 24 h and DCFH-DA (5 *μ*M) was added 30 min before the termination of drug treatment in dark. The cells were then washed in PBS, trypsinized, and resuspended in 3 mL of PBS and the intensity of green fluorescence was immediately read in a fluorescence spectrophotometer (Spectrofluorophotometer RF-5301PC, Shimadzu) (*λ*_ex_ = 488 nm and *λ*_em_ = 525 nm). Results were expressed as arbitrary units of fluorescence per 10^6^ cells.

### 3.6. Statistical Analysis

Descriptive statistical analyses were performed using Excel software (Microsoft Office 2010) for calculating the means and the standard error of the mean. Results were expressed as the mean ± standard deviation (SD). *p* value of less than 0.05 was considered statistically significant.

## 4. Results

### 4.1. Total Phenolic and Flavonoid Content

The antioxidant activity of plant materials was well correlated with the content of their phenolic and flavonoid compounds. Hence the levels of total phenolic (TPC) and flavonoid (TFC) contents were measured. This sample showed high total phenolic (TPC) and flavonoid (TFC) contents as shown in Table 1. The TPC was expressed as gallic acid equivalent (GAE) in *μ*g/mg extract. The TPC is shown in Table 1 with the value of 293.01 ± 12.5 (*μ*gGAE/1 mg extract). TFC was found to be 76.1 ± 1.8 mg QE 1 g extract.

### 4.2. Antioxidant Activity

Antioxidant properties of khat were assessed using various chemical assays with different objectives namely, FRAP, DPPH, and ORAC, respectively. Free radical scavenging is one of the known mechanisms by which antioxidants inhibit lipid oxidation. In Table 1, the scavenging activity of the DPPH radical due to its reduction by extract is illustrated, whereby khat showed IC_50_ of 15.8 ± 0.8 *μ*g/ml for DPPH assay. The ability of the plants extracts to reduce ferric ions was determined using the FRAP assay. An antioxidant capable of donating a single electron to the ferric-TPTZ (Fe(III)-TPTZ) complex would cause the reduction of this complex into the blue ferrous-TPTZ (Fe(II)-TPTZ) complex which absorbs strongly at 593 nm. As Table 1 shows, khat leaves showed very high FRAP value of 4514 ± 100.8 which higher than gallic acid and querciten. Moreover the AUC of 46.1 was observed for ORAC assay.

### 4.3. Effect of Khat on Cell Viability and Nitrite Production

The MTT assay was done to determine the suitable concentration of extract that would not affect cellular viability. As shown in Table 2, following khat treatment, cytotoxicity on RAW 264.7 from 3.75 to 30 *μ*g/ml was not significant. Hence this dose range was used in the nitric oxide determination. The induction of RAW 264.7 cells with LPS caused synthesis and secretion of NO. The extract had showed a dose-related inhibition of NO production in which significant inhibition of 87.5% at 30 *μ*g/ml.

### 4.4. Determination of Reactive Oxygen Species Formation

**Figure 1:**
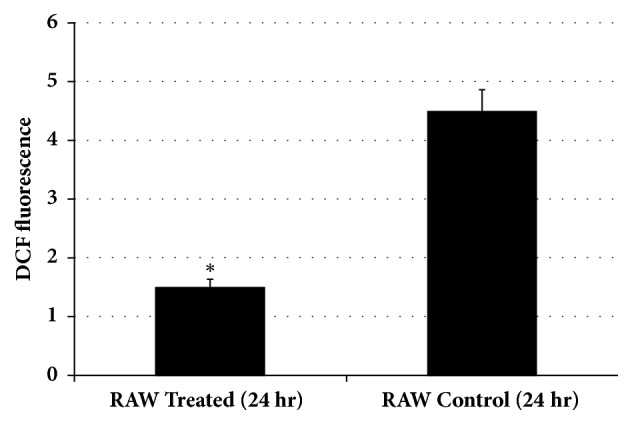
Khat inhibited intracellular ROS generation in RAW cells. All results are expressed as arbitrary units of the fluorescence intensity per 10^6^ cells and reflect mean ± SE, ^*∗*^*p* < 0.01; compared with untreated group.

The intercellular ROS levels in khat-treated RAW cells were investigated through oxidation-sensitive DCF fluorescence intensity. The nonfluorescent H2 DCFDA is easily permeabilized through cell membrane and got oxidized into highly fluorescence DCF in the presence of ROS. The increase in fluorescence intensity is directly proportional to the increased ROS formation in the cell. Treatment of cells with khat caused a highly significant decrease in ROS formation as evidenced by decrease in green fluorescence intensity of treated cells which indicates inhibited ROS formation at 24 hours (Figure 1).

## 5. Discussion

Khat induces various symptoms including anorexia, insomnia, euphoria, GIT symptoms, and sexual impotence. Toxic effects of crude khat extract have been studied over the last 25 years using* in vivo *and* in vitro* models [14, 16, 23]. Genotoxic effects were also investigated using Bactria, peripheral blood, and mice bone marrow [24]. However, there is no data on the association of the toxic properties of khat crude extract and its phytochemical ingredients. Our previous research has shown a considerable amount of polyphenol content. Unwanted effects of polyphenols have been assessed principally in laboratory-based research. Moreover, we have previously shown that epigallocatechin and epigallocatechin gallate are the major compounds in samples of khat obtained from Ethiopia and Yemen [20]. It is recognized, for instance, that definite polyphenols may have genotoxic/carcinogenic properties [25]. Many articles have reviewed the health impact of khat consumption; however the role of free radicals in the pathogenesis associated with short- and long-term consumption of khat is absent in the literature. As free radicals and antioxidants congregate across different mechanisms in normal physiological mechanisms and in disease, various researches were done to expose the role of endogenous free radicals and the mechanism of cellular injury related to khat consumption [26]. Therefore, the current study was conducted to understand the association between phenolic rich extract of khat and its induced toxicity and anti-inflammatory properties.

Several classes of flavonoid and phenolic show antioxidant activity toward a variety of readily oxidizable compounds [27]. They exist widely in the plant kingdom as an important part of the diet because of their effects on human nutrition. The phenolic and flavonoids can modulate lipid peroxidation involved in atherogenesis, thrombosis, and carcinogenesis, and their known properties include free radical scavenging, strong antioxidant activity, inhibition of hydrolytic and oxidative enzymes, and anti-inflammatory action [28]. In order to measure the antioxidant capacity of the khat extract obtained from Saudi Arabia, the total phenolic (TPC) and flavonoid (TFC) contents were measure. Our sample showed high total phenolic (TPC) and flavonoid (TFC) contents as shown in Table 1. Hydroalcoholic extract of khat leaves was reported earlier to possess very high hydrophilic antioxidant capacity, comparable to that of the tea leaves [15, 29].

In order to measure the total antioxidant capacity of khat extract, FRAP, DPPH, and ORAC were utilized. Free radical scavenging activity of khat using DPPH has been reported earlier [30]. This method is based on scavenging of DPPH through the addition of a radical species or antioxidant that decolourizes the DPPH solution [31]. The degree of colour change is proportional to the concentration and potency of the antioxidants. We observed an IC_50_ of 15.8 ± .8 *μ*g/ml for DPPH assay. Results from this assay shows that the khat extract contains phytochemical constituents that are capable of donating hydrogen to a free radical to scavenge the potential damage. The current study is the first of its kind to assess the antioxidant activity of khat using FRAP and ORAC assays. The ORAC assay measures the antioxidant scavenging function against peroxyl radical induced by AAPH. Fluorescein is used as a fluorescent probe. The loss of fluorescence of fluorescein is an indication of the extent of damage from its reaction with the peroxyl radical [21].

Normal cellular metabolism produces reactive oxygen (superoxide anion, hydrogen peroxide, hydroxyl radical, and organic peroxides) and nitrogen species and these play critical roles in activating the downstream of signaling pathways in animal cells which alter the intra- and extracellular metabolic activities [32]. This inflammatory/oxidative dynamic imbalance prompts a harmful circle, which can damage healthy surrounding healthy cells, which after a lengthy period of time may cause carcinogenesis [33]. This leads to the explanation of oral cancer induced by khat chewing [34]. The inhibition of NO production by khat derived in the current study may be due to the decrease of inducible nitric oxide synthase activity [35]. Moreover, MTT assay showed that khat induced cytotoxic effects on RAW 264.7 macrophages. In our previous research, khat's adverse effects were proven* in vivo *and* in vitro *[14–16]. This indicates that khat-induced anti-inflammatory properties are accompanied by concurrent toxic effects. Higher doses of crude khat extract have immune suppressing property [36]. Administration of khat was reported to induce nephrotoxicity in rats through the induction of oxidative stress by depleting antioxidative mechanisms or by enhancing prooxidant ingredients of tissues, causing renal damage [37].

The term ROS is used for short-lived diffusible entities such as hydroxyl (^*∙*^OH), alkoxyl (RO^*∙*^), or peroxyl (ROO^*∙*^) radicals and for some radical species of medium lifetime such as superoxide (O_2_^ ^^∙^) or nitroxyl radical (NO^*∙*^) [38]. To explore the possibility of ROS mediated cell death, DCFH-DA assay was performed with IC_50_ concentration of khat. One of the major objectives of this study was to find out the progression of cellular signaling events that happen after modulation of the cellular redox state in cells. So, our study will provide for the first time practical evidence on the role of ROS inhibition in cell death in khat phenolic rich extract. Previous reports have shown that khat was able to induce apoptosis in H9c2 cardiac and Madin-Darby bovine kidney cell lines [16, 17]. According to the best of our knowledge and belief, this is the first* in vitro* study demonstrating the relationship between ROS and cell death for khat.

## 6. Conclusion

Long-term khat chewing has negative effects on the oral mucosal tissues. Khat appears to be capable of disturbing the delicate equilibrium between damaging and protective mechanisms of a cell that is essential for optimal activity, thereby producing oxidative damage. Current findings revealed that khat possesses high phenolic and flavonoid contents with oxygen and nitrogen free radicals scavenging activities. It also modulates the inflammatory process of the murine monocytic macrophages cell line (RAW 264.7) with potential cytotoxic effects. According to the best of our knowledge and belief, this is the first* in vitro* study demonstrating the relationship between ROS and cell death for khat. Current findings warrant further* in vivo* studies.

## Figures and Tables

**Table 1:** Total phenolic and flavonoid contents and antioxidant activities of khat.

Samples	TPC (*µ*gGAE/1 mg extract)	TFC (mg QE 1 g extract)	FRAP value	DPPH scavenging activity (IC_50_) *µ*g/ml	ORAC	
					AUC	Equivalent conc. Trolox @ 100 *μ*g/mL
Khat	293.01 ± 12.5	76.10 ± 1.8	4514 ± 100.8	15.8 ± 1.8	46.1	900.16 ± 0.91
Gallic acid			2645.6 ± 124.8	7.18 ± 0.98		
Querciten			1925.2 ± 29.8	8.2 ± 0.28	12.4	198.2 ± 0.58

All samples and positive control were done in triplicates (*n* = 3). AUC: area under the curve was calculated for the sample, standard, and the positive controls from the equation mentioned earlier.

**Table 2:** Inhibitory activities of khat on the activated NO production in RAW 264.7 macrophages. Quercetin was used as a standard drug.

Khat Concentration (*μ*g/mL)	NO (*μ*M)	% NO inhibition	% Cell viability
30	0.78 ± 0.21	87.5	80
15	1.3 ± 0.05	80	78
7.5	2.9 ± 0.09	54	70
3.75	5.73 ± 0.03	8	68
Control	6.27 ± 0.28		
